# Direct Observation of Contact Ion-Pair Formation in La^3+^ Methanol Solution

**DOI:** 10.1021/acs.inorgchem.2c02932

**Published:** 2022-10-18

**Authors:** Paola D’Angelo, Valentina Migliorati, Alice Gibiino, Matteo Busato

**Affiliations:** Department of Chemistry, University of Rome “La Sapienza”, P.le Aldo Moro 5, 00185 Rome, Italy

## Abstract

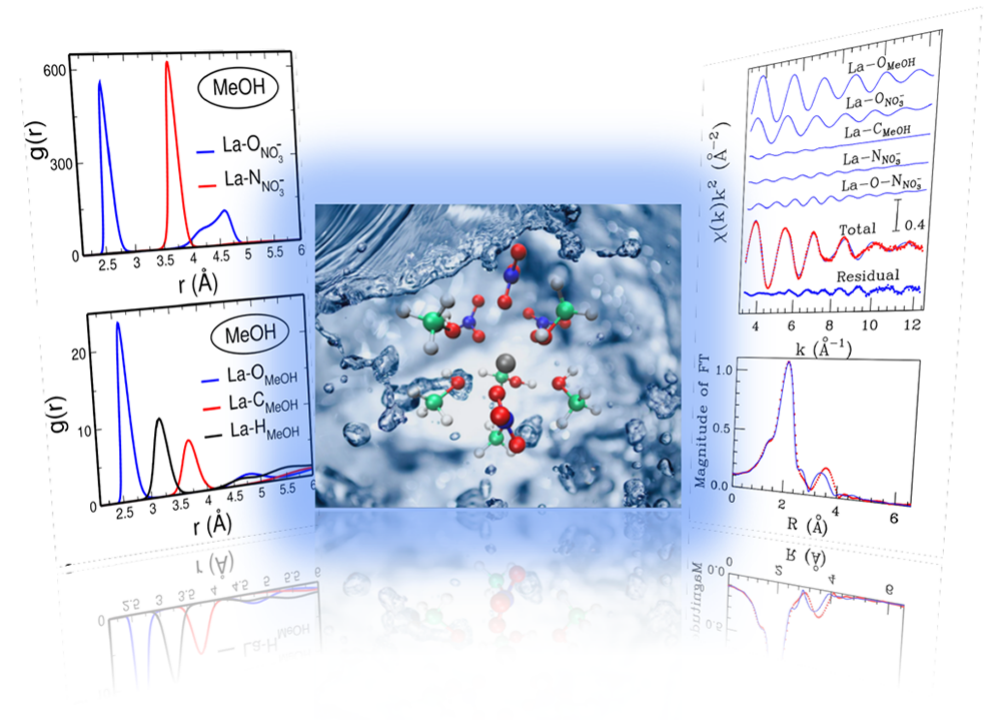

An approach combining molecular dynamics (MD) simulations and X-ray absorption spectroscopy (XAS) has been used to carry out a comparative study about the solvation properties of dilute La(NO_3_)_3_ solutions in water and methanol, with the aim of elucidating the still elusive coordination of the La^3+^ ion in the latter medium. The comparison between these two systems enlightened a different behavior of the nitrate counterions in the two environments: while in water the La(NO_3_)_3_ salt is fully dissociated and the La^3+^ ion is coordinated by water molecules only, the nitrate anions are able to enter the metal first solvation shell to form inner-sphere complexes in methanol solution. The speciation of the formed complexes showed that the 10-fold coordination is preferential in methanol solution, where the nitrate anions coordinate the La^3+^ cations in a monodentate fashion and the methanol molecules complete the solvation shell to form an overall bicapped square antiprism geometry. This is at variance with the aqueous solution where a more balanced situation is observed between the 9- and 10-fold coordination. An experimental confirmation of the MD results was obtained by La K-edge XAS measurements carried out on 0.1 M La(NO_3_)_3_ solutions in the two solvents, showing the distinct presence of the nitrate counterions in the La^3+^ ion first solvation sphere of the methanol solution. The analysis of the extended X-ray absorption fine structure (EXAFS) part of the absorption spectrum collected on the methanol solution was carried out starting from the MD results and confirmed the structural arrangement observed by the simulations.

## Abstract 2

The formation of contact ion pairs between the La^3+^ ions and the nitrate anions has been demonstrated in diluted methanol solution using a combined approach using Molecular Dynamics simulations and X-ray absorption spectroscpy.

## Introduction

Lanthanide 3+ (Ln^3+^) cations are of great scientific and technological importance, being involved in many applications including medical diagnosis, organic synthesis, catalysis, nuclear waste management, and liquid–liquid extraction.^1−4^ Moreover, they are used as surrogates for the study of the actinide ions because of their lower toxicity and radio-inactivity.^5^ The dissolution of Ln^3+^ ions in solvents with different polarity is widely employed for the separation of these species in nuclear power technology and industrial processes.^4^ Lanthanides constitute about one-third of the fission products from spent nuclear fuel rods, which, after processing, provide a range of valuable products. Understanding such processes requires a detailed and quantitative knowledge of the solution chemistry of the Ln^3+^ ions, and one very important aspect is their tendency to form ion pairs in solution with inorganic counterions that are commonly employed in nuclear and other types of processing. The quantification of ion–ion interactions, which is fundamental for a reliable chemical speciation modeling, is problematic because they are often not very strong and have a tendency to form solvent-separated species.^6^

The influence of counterions on the coordination structure and dynamics of Ln^3+^ ions is a topic greatly debated in the literature and, in this respect, water has been the most studied solvent.^7−13^ In highly concentrated aqueous solutions (>1 M) contact ion pairs do form due to the low number of water molecules that are not sufficient to complete the first and second hydration shells of the ions. For more dilute solutions the effect of the counterions strongly depends on their chemical nature. Perchlorate (ClO_4_^–^) and triflate (trifluoromethanesulfonate, TfO^–^) are considered weakly complex-forming anions, and it is well established that they do not enter the Ln^3+^ first hydration shell,^8−15^ while for chloride ions an inner-sphere complexation has been found to occur only at high salt concentrations.^10,12,13,16−19^

As concerns the nitrate anions, an unambiguous determination of their ability to form contact ion pairs with the Ln^3+^ ions is still lacking. Indeed, according to some studies nitrate coordinates such cations in their first coordination sphere,^10,12,20−24^ while other investigations claim that nitrate does not form inner-sphere complexes.^25−29^ On the other hand, the knowledge of the structure of lanthanide nitrates in solution is fundamentally important for the development of extraction technologies for the separation of lanthanides and minor actinides in the processing of high-level waste, which is one of the most urgent tasks in the modern nuclear fuel cycle.^30^ Indeed, nitrate takes part in spent nuclear fuel reprocessing as well as in nuclear waste, and complexation with nitrate could alter the speciation and the chemical behavior of lanthanides in the reprocessing processes.^30^ In this paper, we focus on the lanthanum nitrate salt, and in order to provide a reliable structural description of the complexes formed by the La^3+^ ion in solution, we applied a powerful synergic approach combining molecular dynamics (MD) simulations and X-ray absorption spectroscopy (XAS) as previously done for several liquid systems.^31−39^

From an experimental point of view, Ln^3+^ complexation with perchlorate, halide, nitrate, and triflate has been studied with different techniques such as XAS,^8,9,16^^17^O and ^19^F NMR,^11,40^ Raman,^12,13^ UV–visible,^10^ and time-resolved laser-induced fluorescence spectroscopies.^17^ However, although these techniques provide reliable structural or dynamic information, a thorough microscopic description of the short-range structure of Ln^3+^ salts in aqueous and organic solvents is still lacking, and in this respect, the XAS technique is ideally suited to provide accurate short-range structural information around a selected ion.

While much effort has been devoted to unveil the coordination structure of lanthanide ions in water, their solvation properties in nonaqueous solvents have received less attention.^41^ Among the organic solvents, methanol shows intriguing characteristics, as it is the simplest organic compound having both a hydrophobic and hydrophilic group. Moreover, methanol is a close analogue to water, because of the presence of the hydroxyl group, and it is able to form a strong network of hydrogen bonds, which is responsible for many properties of the bulk liquid. Due to the similar nature of ion–water and ion–methanol interactions, methanol is the ideal solvent to shed light on the complexing ability of nitrate anions toward the La^3+^ ion in the presence of solvents with slightly different solvation capacity. It is thus very interesting to understand if La^3+^ forms similar solvation complexes in water and methanol, as previously found for transition metal ions such as Zn^2+^^42,43^ or for alkaline ions such as Na^+^.^44^ Note that, from a theoretical point of view, a variety of solvation structures have been obtained for the La^3+^ ion in liquid media: 9-fold clusters in pure water,^45^ [LaNO_3_(H_2_O)_7_]^2+^ species with bidentate nitrate ions in La(NO_3_)_3_ aqueous solutions,^46^ 7-fold and 8-fold ionic structures in molten LaCl_3_,^47^ and 10- and 12-fold complexes in ethylammonium nitrate/methanol mixtures,^48^ to cite a few. Moreover, it is very interesting to investigate the nitrate coordination mode toward the La^3+^ cations in methanol. In solid state structures the dominant coordination of nitrate is the bidentate one,^49^ while in water the monodentate binding mode was shown to be favored.^10,22,29^ To shed light on all these points, we have decided to carry out an MD and XAS investigation on aqueous and methanol solutions of the La^3+^ nitrate salt in dilute conditions (0.1 M). This powerful combined approach allowed us to shed light on the solvation properties of the La^3+^ ion in methanol and also to study the formation of contact ion pairs between nitrate and La^3+^ in the two solvents.

## Methods

### Molecular Dynamics

Classical MD simulations of 0.1 M methanol and aqueous solutions containing La(NO_3_)_3_ have been carried out by means of the Gromacs package.^50^ The SPC/E model^51^ and OPLS/AA force field^52^ have been used for the water and methanol molecules, respectively, while the force field parameters for the nitrate anions were taken from Lopes and Padua.^53^ The Lennard–Jones parameters for the La^3+^ cations were those developed by us in ref (54). Mixed Lennard–Jones terms for the different atom types were obtained from the Lorentz–Berthelot combination rules, with the exception of those related to the La–O_*NO*_3_^–^_ interaction in methanol (where O is the oxygen atom of the nitrate anion) that were developed *ad hoc* to describe the La(NO_3_)_3_ salt dissolved in EAN solution,^55^ while in water they were taken from the values used to describe the La–O interaction with water molecules.^54^ The systems were composed of 5 La^3+^ cations, 15 nitrate anions, and either 2775 water or 1227 methanol molecules in a cubic box, created by randomly assigning initial positions to all ions and molecules, and then compressed and equilibrated under NPT conditions in order to obtain the starting configurations. The NPT simulations have been carried out at 1 atm and 300 K for 5 ns, using the Nosé–Hoover thermostat and barostat with 0.5 and 2.0 ps relaxation times, respectively. The simulations have been carried out in the NVT ensemble at 300 K for a total time of 20 ns, after a 10 ns equilibration run, using a 1 fs time step. The lengths of the simulation box edges are 43.87 and 43.80 Å for the aqueous and methanol solutions, respectively. The Nosé–Hoover thermostat,^56,57^ with a relaxation constant of 0.5 ps, was used to control the system temperature. Long-range interactions have been evaluated by the particle-mesh Ewald method,^58^ while the cutoff of the nonbonded interactions was set to 12 Å. Periodic boundary conditions have been applied in order to simulate bulk material.

The structural properties of the simulated solutions have been characterized by calculating the radial (*g*(*r*)’s) and combined (CDFs) distribution functions. To have a quantitative description of the coordination around the La^3+^ cations, the *g*(*r*)’s have been modeled with Γ-like functions depending on four parameters: the average distance *R*, the coordination number *N*, the Debye–Waller factor σ^2^, and the asymmetry index β. This function is defined in a wide interval of positive and negative asymmetry values and falls in the Gaussian limit for β → 0.^59−62^

### X-ray Absorption Measurements

XAS measurements were carried out on La(NO_3_)_3_ 0.1 M solutions in water and methanol. La(NO_3_)_3_·*n*H_2_O (Sigma-Aldrich, 99.5%) was dried under Ar flux at 200 °C for 2 h to remove water. The solutions were then prepared by dissolving a stoichiometric amount of the salt in respectively Milli-Q water or anhydrous MeOH (Sigma-Aldrich). La K-edge spectra were acquired at room temperature in transmission mode at the BM23 beamline of the European Synchrotron Radiation Facility (ESRF). The data were collected with a Si(311) double-crystal monochromator with the second crystal detuned by 20% for harmonic rejection. Cells with Kapton windows and 2 mm Teflon spacers were filled with the sample, while at least three spectra were collected and averaged for each solution.

### EXAFS Data Analysis

The analysis of the EXAFS spectrum collected on the La(NO_3_)_3_ solution in methanol was carried out with the GNXAS code.^63,64^ Amplitudes and phase shifts have been calculated from clusters obtained from the MD simulations including the La^3+^ ion and ten methanol molecules, in the framework of the muffin-tin approximation, employing advanced models for the exchange-correlation self-energy (Hedin-Lundqvist), which intrinsically take into account the photoelectron inelastic losses in the final state.^65^ Theoretical signals associated with *n*-body distribution functions have been calculated in accordance with the multiple-scattering (MS) theory and summed in order to obtain the total theoretical contribution. Two-body single scattering (SS) theoretical signals have been calculated to take into account the first shell oxygen atoms of the methanol molecules (La–O_*MeOH*_) and of the nitrate anions (La–O_*NO*_3_^–^_), while the La–C_*MeOH*_ and La–N_*NO*_3_^–^_ signals have been calculated for the second-shell carbon and nitrogen atoms, respectively, starting from the structural parameters determined from the MD *g*(*r*)’s. In addition, the MS signal describing the three-body La–O–N_*NO*_3_^–^_ distribution with a bond angle of 180° was also included. Differently, the La–O–C_*MeOH*_ MS term was found to possess a negligible amplitude due to the bent configuration assumed by the coordinating methanol molecules.

Least-squares minimizations have been carried out on the raw data directly, without preliminary background subtraction and Fourier filtering, by optimizing all the structural parameters starting from the values obtained from the MD simulation of the same system. Nonstructural parameters have been also optimized, namely the K-edge ionization energy *E*_0_, and the energy position and amplitude of the double-electron excitation channels. The inclusion of the double-electron excitations allowed us to keep the amplitude reduction factor *S*_0_^2^ constrained to 1.0.

## Results and Discussion

### MD Results

**Figure 1:**
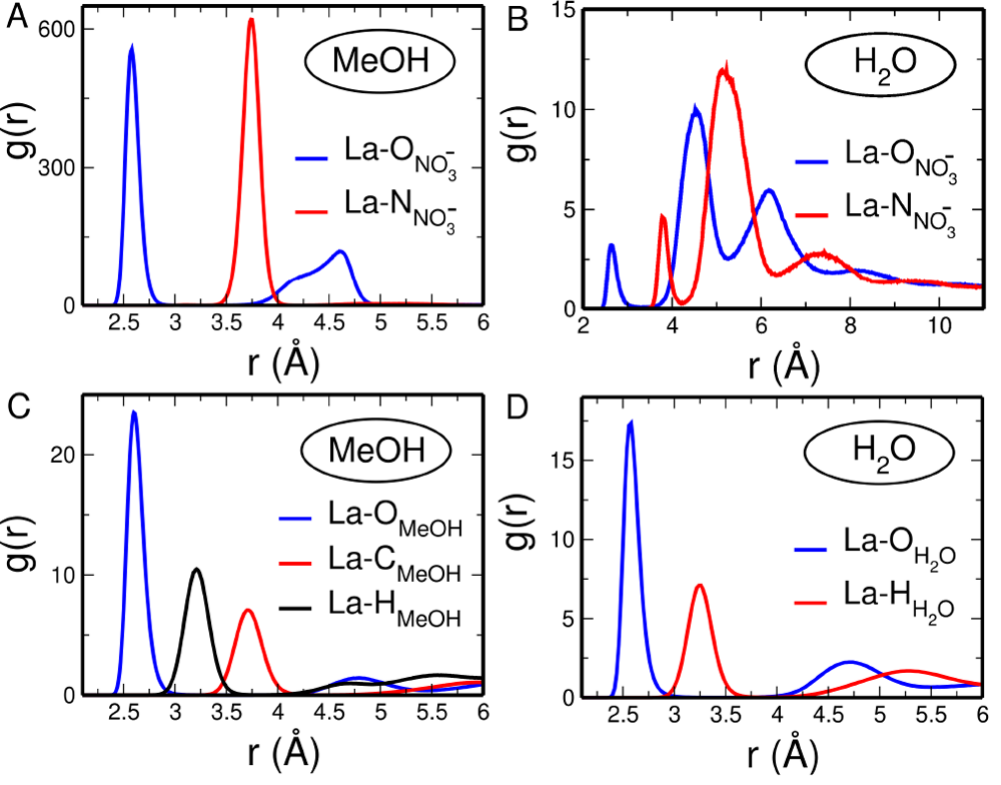
(A) La–O_*NO*_3_^–^_ (blue line) and La–N_*NO*_3_^–^_ (red line) radial distribution functions *g*(*r*)’s, where O and N are the oxygen and nitrogen atoms of the nitrate anions, respectively, calculated from the MD simulation of the 0.1 M La(NO_3_)_3_ methanol solution. (B) La–O_*NO*_3_^–^_ (blue line) and La–N_*NO*_3_^–^_ (red line) radial distribution functions *g*(*r*)’s, where O and N are the oxygen and nitrogen atoms of the nitrate anions, respectively, calculated from the MD simulation of the 0.1 M La(NO_3_)_3_ aqueous solution. (C) La–O_*MeOH*_ (blue line), La–C_*MeOH*_ (red line), and La–H_*MeOH*_ (black line) *g*(*r*)’s, where O, C, and H are the oxygen, carbon, and hydrogen atoms of the methanol molecules respectively, calculated from the MD simulation of the 0.1 M La(NO_3_)_3_ methanol solution. (D) La–O_*H*_2_*O*_ (blue line) and La–H_*H*_2_O_ (red line) *g*(*r*)’s, where O and H are the oxygen and hydrogen atoms of the water molecules respectively, calculated from the MD simulation of the 0.1 M La(NO_3_)_3_ aqueous solution.

The first question that we address here is whether the La(NO_3_)_3_ salt forms inner-sphere complexes in water and in methanol. To this end, we have carried out MD simulations of both the aqueous and the methanol solution containing La(NO_3_)_3_ (0.1 M), as described in detail in the Methods section. In order to investigate the La(NO_3_)_3_ complexation properties, we have calculated from the MD trajectories the *g*(*r*)’s between the La^3+^ cations and the oxygen atom of the nitrate anions (La–O_*NO*_3_^–^_). In aqueous solution, no nitrate anions have been found in direct contact with the La^3+^ cations as the La–O_*NO*_3_^–^_*g*(*r*) does not show any significant peak in the short distance range (Figure 1B), indicating that the La(NO_3_)_3_ salt is fully dissociated in water, in agreement with previous XAS investigations.^29^ Note that the low-intensity peak at 2.65 Å is negligible since the corresponding La–O_*NO*_3_^–^_ coordination number is 0.04. Even if no nitrate anions are present in the La^3+^ first shell complex, they are found in the second solvation shell of the La^3+^ ion, as shown by the presence of a peak in the La–O_*NO*_3_^–^_*g*(*r*) with the maximum position at 4.52 Å. By calculating the *g*(*r*) between the La^3+^ cations and the nitrogen atom of the nitrate anions La–N_*NO*_3_^–^_ (Figure 1B) and integrating the second peak of such *g*(*r*), we obtain a La–N_*NO*_3_^–^_ second shell coordination number of 0.8, indicating that almost one nitrate anion is found in the second coordination shell of each La^3+^ cation. At variance with the water case, the La–O_*NO*_3_^–^_*g*(*r*) calculated from the simulation of the methanol solution (Figure 1A) shows a first shell peak with an average distance at 2.60 Å and a coordination number of 4.1 obtained after the fitting procedure with a Γ-function (Table 1). This result shows that nitrate anions in methanol enter the La^3+^ first solvation shell, by forming an inner-sphere complex, at variance with their behavior in aqueous solution. We can therefore state that the nitrate anions have a great affinity toward the La^3+^ cations in methanol, which makes them able to coordinate the La^3+^ ion also in dilute solution where the solvent molecules are present in great excess. The different coordination ability of the nitrate anion in the two solvents can be explained on the basis of the weaker solvation ability of methanol as compared to water.

**Table 1:** Coordination Number *N*, Average Distance *R*, Debye–Waller Factor σ^2^, and Asymmetry Parameter β Obtained from the Fit of the *g*(*r*) First Peak with a Γ-like Function, Calculated from the MD Simulations of the 0.1 M La(NO_3_)_3_ Methanol Solution

	*N*	*R* (Å)	σ^2^ (Å^2^)	β
La–O_*NO*_3_^–^_	4.1	2.60	0.005	0.5
La–N_*NO*_3_^–^_	4.0	3.75	0.007	0.0
La–O_*MeOH*_	5.8	2.63	0.007	0.6
La–H_*MeOH*_	5.9	3.22	0.014	0.1
La–C_*MeOH*_	5.8	3.73	0.017	0.1

**Figure 2:**
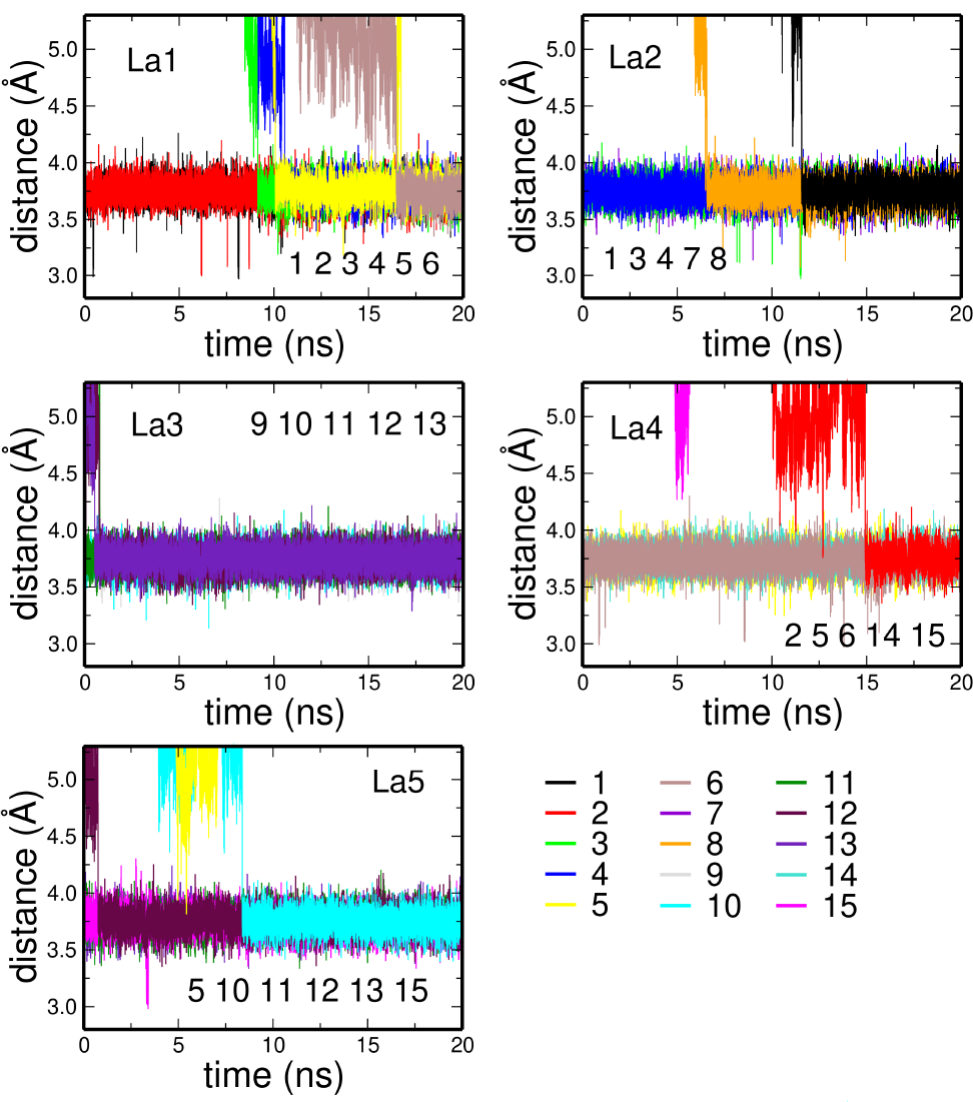
Time evolution of the distances between the La^3+^ cations and the nitrogen atom of the nitrate anions La–N_*NO*_3_^–^_ calculated from the MD simulation of the 0.1 M La(NO_3_)_3_ methanol solution. The distances have been calculated for each La^3+^ cation and each nitrate anion which enters the La^3+^ first solvation shell during the entire simulation time. The indexes of all the anions entering the first shell complex of the different La^3+^ cation in the course of the entire simulation are specified for clarity.

It is interesting to point out that the number of nitrate anions in the La^3+^ first solvation shell is higher than the ratio between La^3+^ and NO_3_^–^ which is 1:3. This is due to a dynamical equilibrium where some of the nitrate anions present in the first shell of a given La^3+^ cation at a given time enter the first shell of another La^3+^ ion at another time. Moreover, some nitrate anions can be shared between two different La^3+^ cations, forming a bridge structure, and such behavior can be clearly observed by looking at the time evolution of the distances between the La^3+^ cations and the nitrogen atom of the nitrate anions La–N_*NO*_3_^–^_ that have been calculated from the MD simulation of the methanol solution (see Figure 2). Note that this analysis has been carried out for each La^3+^ cation and each nitrate anion that enter the La^3+^ first solvation shell during the entire simulation time.

An open question concerning the nitrate coordination of lanthanide ions is whether the nitrate ions act as monodentate or bidentate ligands. It is therefore interesting to investigate the nitrate coordination mode toward the La^3+^ cations in methanol by calculating the *g*(*r*)’s between the La^3+^ ions and the N atom of the nitrate anions La–N_*NO*_3_^–^_ (Figure 1A). As it can be seen, a single peak is observed, with an average distance of 3.75 Å and a coordination number of 4.0 (Table 1), showing that a single coordination mode is present in methanol, namely the monodentate one, in line with previous results obtained for lanthanide aqueous solutions.^10,22,29,66^

Besides interacting with the nitrate anions, the La^3+^ ion in methanol forms short-range interactions with the solvent molecules, as shown by the *g*(*r*) calculated between the La^3+^ cations and the methanol oxygen atom (La–O_*MeOH*_) which is reported in Figure 1C. The La–O_*MeOH*_*g*(*r*) first shell peak presents a maximum at an average distance of 2.63 Å and a first shell coordination number of 5.8 (Table 1). We can notice that the La–O_*MeOH*_ distance tends to be similar to the La–O_*NO*_3_^–^_ one, and that the La^3+^ solvation shell is mainly constituted of methanol molecules. To gain deeper insight into the methanol arrangement in the La^3+^ coordination shell, it is useful to calculate also the La–C_*MeOH*_ and La–H_*MeOH*_*g*(*r*)’s between the La^3+^ cations and the methanol carbon and hydroxyl hydrogen atom, respectively (Figure 1C). The *g*(*r*)’s show very sharp and separated first peaks followed by depletion zones, indicating the existence of a preferential orientation of the methanol molecules in the La^3+^ first coordination sphere, which is rather ordered and structured.

In aqueous solution, where no nitrate ions enter the La^3+^ coordination complex, the La^3+^ ion forms strong interactions with the water molecules. Indeed, the *g*(*r*)’s between La^3+^ and the water oxygen and hydrogen atoms (La–O_*H*_2_*O*_ and La–H_*H*_2_*O*_, respectively) show very sharp and structured first shell peaks (Figure 1D), and the neat separation between the La–O_*H*_2_*O*_ and La–H_*H*_2_*O*_*g*(*r*) first peaks indicates that also the water molecules are strongly oriented due to the electrostatic field of the La^3+^ ion. The average distance of the La–O_*H*_2_*O*_*g*(*r*) first peak maximum is 2.61 Å, showing that the La^3+^ cation coordinates the oxygen atoms of methanol, water, or nitrate ions at similar distances. Moreover, the La–O_*H*_2_*O*_ first shell average coordination number is 9.5, thus suggesting that the La^3+^ solvation shell contains a larger number of ligands in methanol solution as compared to water. Indeed, the La^3+^ total coordination number in methanol calculated as the sum of the La–O_*NO*_3_^–^_ and La–O_*MeOH*_ coordination numbers is 9.9.

**Figure 3:**
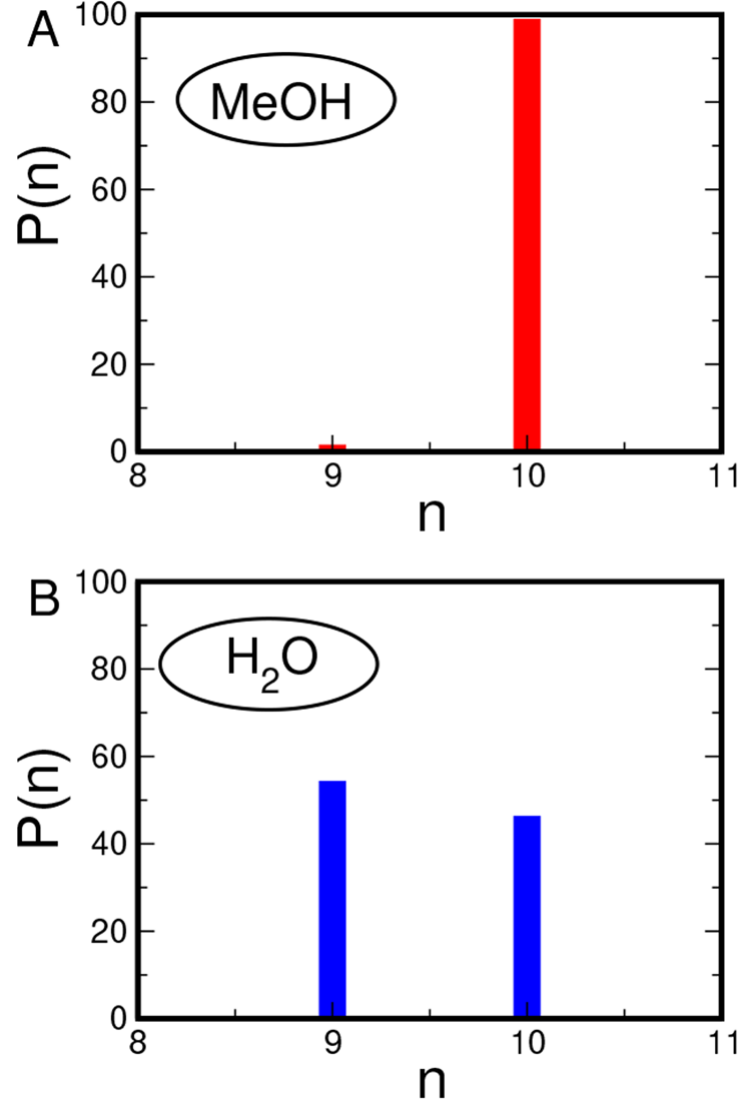
(A) Coordination number distribution (*P*(*n*)) of the sum of the oxygen atoms of the methanol molecules and nitrate anions in the La^3+^ first solvation shell calculated from the MD simulation of the 0.1 M La(NO_3_)_3_ methanol solution. (B) *P*(*n*) of the oxygen atoms of the water molecules in the La^3+^ first solvation shell calculated from the MD simulation of the 0.1 M La(NO_3_)_3_ aqueous solution.

Deeper insight into this behavior can be obtained by defining a total instantaneous coordination number *n* of La^3+^ as the number of atoms at a distance from the cation shorter than 3.30 Å, and analyzing its variation along the simulations. If one considers the La^3+^ first shell complex constituted of oxygen atoms either of methanol or nitrate, almost 100% of La^3+^ ions present 10 first neighbors (Figure 3A), while in water a dominant percentage of La^3+^ coordination complexes contains 9 solvent molecules. Note that the percentage of 10-fold structures calculated from our simulation of the aqueous solution is overestimated as compared to the results obtained for the La^3+^ ion in water from both the EXAFS analysis and the MD simulations carried out by including explicit polarization.^67^ This is due to the use of Lennard–Jones potentials without explicit polarization in the MD simulation, as already pointed out in the literature.^54^

**Figure 4:**
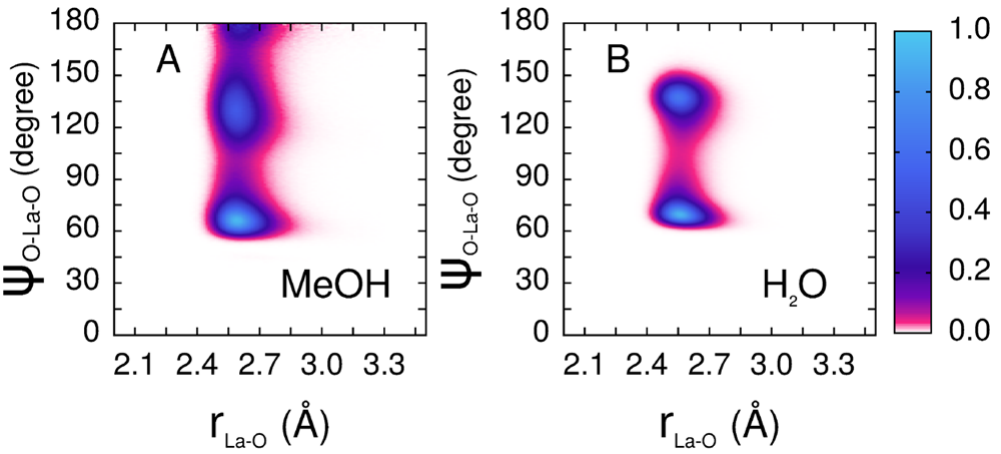
(A) CDFs between the La–O distances and O–La–O angles evaluated for the 10-fold configurations only extracted from the MD simulation of the 0.1 M La(NO_3_)_3_ methanol solution. O is the oxygen atom of either methanol molecules or of nitrate anions belonging to the La^3+^ first coordination shell. (B) CDFs between the La–O distances and O–La–O angles, where O is the oxygen atom of the water molecules belonging to the La^3+^ first coordination shell, evaluated for the 9-fold configurations only extracted from the MD simulation of the 0.1 M La(NO_3_)_3_ aqueous solution.

In order to identify the global geometry of the coordination polyhedra formed by the La^3+^ ions in methanol and aqueous solution, we have calculated the CDFs between the La–O distances and the O–La–O angles.^68^ In the case of water, O is the water oxygen atom, while for the methanol solution, O is the oxygen atom of either methanol molecules or nitrate anions belonging to the first coordination shell of the La^3+^ ion. This analysis has been carried out for the dominant configurations found in the studied systems, namely for the La^3+^ 10-fold and 9-fold structures in methanol and aqueous solutions, respectively. The CDF calculated for the La^3+^ 10-coordinated first shell clusters formed in methanol (Figure 4A) shows three peaks at about 65°, 130°, and 180°, pointing to the existence of a bicapped square antiprism (BSAP) geometry of the ten oxygen atoms surrounding the La^3+^ ion. In order to better visualize the origin of the three peaks obtained in the CDF analysis, we have shown in Figure S1 a model of the ideal 10-fold bicapped square antiprism polyhedron with each pair of atoms involved, together with the central La^3+^ ion, in the angles calculated in the CDF analysis. The different couples of atoms are colored with different colors and can be grouped as follows. The angles involving capped atom–atom in the neighboring square (magenta circles), neighboring atoms in a square (black circles), and atom in a square-neighboring atom in the other square (orange circles) contribute to the peak at about 65°; the angles involving atoms on the opposite sides of a square (yellow circles), atom in a square-atom on the opposite side of the other square (gray circles), and capped atom–atom in the opposite square (green circles) generate the 130° peak; capped atom–capped atom (blue circles) produces instead the peak at 180°.

As concerns the 9-fold first shell complexes formed by La^3+^ in water, two peaks at O–La–O angles of 70° and 135° are found in the corresponding CDF (Figure 4B). In this case, the form of the distribution with the characteristic L-shape of the low angle peak is consistent with a tricapped trigonal prism (TTP) geometry of the first shell cluster, in agreement with the results reported in the literature for light Ln^3+^ ions.^54,69−71^

**Figure 5:**
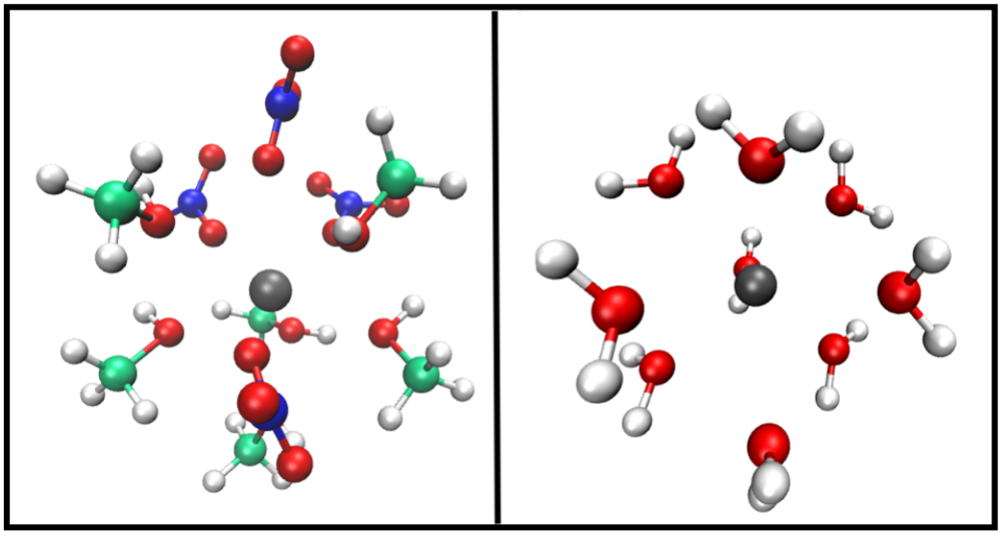
MD snapshots showing the coordination environment of the La^3+^ ion in methanol (A) and in aqueous solutions (B) containing the La(NO_3_)_3_ salt. Carbon, nitrogen, oxygen, hydrogen and La atoms are represented in green, blue, red, white and dark gray, respectively.

Two representative MD snapshots showing the different coordination environments of the La^3+^ ion in water and in methanol can be observed in Figure 5. It is well-known that, at variance with transition metal ions for which the coordination number and the geometry of the coordination complexes are determined by the overlap of the metal and ligand orbitals, the solvation chemistry of lanthanide ions is primarily due to electrostatic interactions. As a consequence, they display many different coordination structures, with a coordination number in solid compounds ranging from 3 to 12.^6^ The driving force for the formation of a peculiar molecular arrangement is the maximization of electrostatic forces and the minimization of the repulsion among the ligands belonging to the lanthanide first solvation shell. Moreover, since the solvation complexes are not in vacuum but immersed in the solution, the maximization of the interactions with the solvent molecules belonging to the lanthanide second solvation shell plays an additional role in determining the overall structure. On the basis of our results, the La^3+^ ion in methanol solution reaches the ideal compromise among all of the different forces in play by forming a 10-fold complex containing both methanol molecules and nitrate anions, while in aqueous solution it prefers to bind only water molecules forming a 9-fold coordination structure.

### XAS results

**Figure 6:**
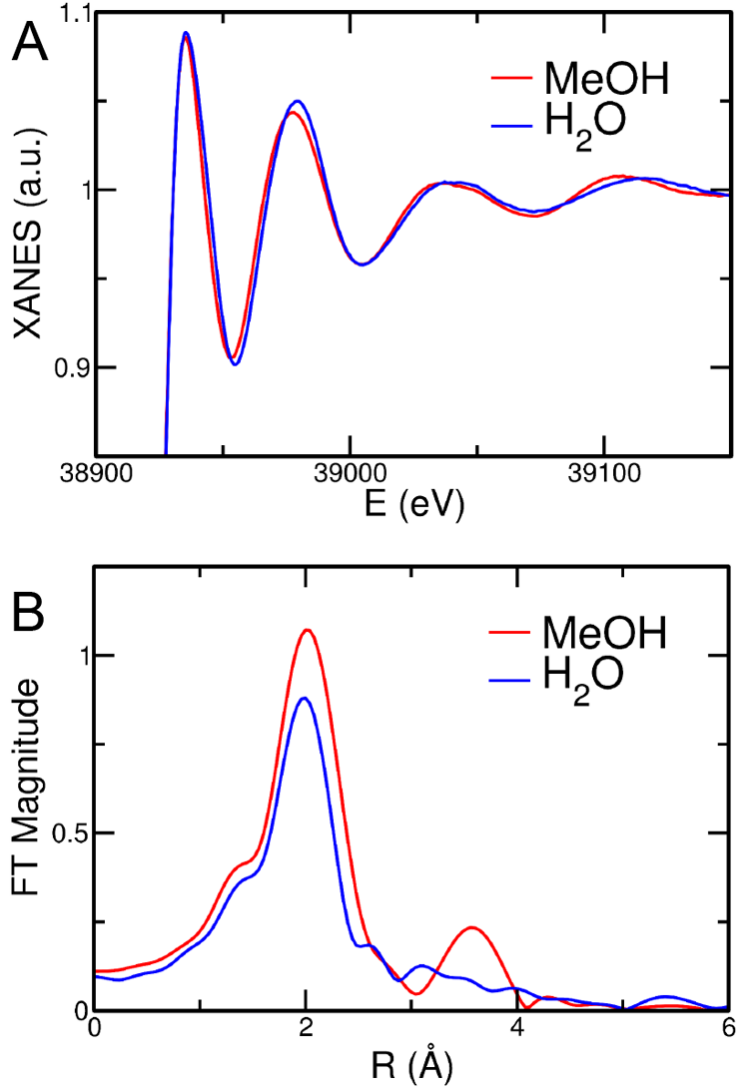
La K-edge normalized XANES experimental spectra (A) and corresponding nonphase shift corrected FTs (B) collected for 0.1 M La(NO_3_)_3_ solutions in water (blue line) and methanol (red line).

To obtain an experimental confirmation of the results obtained from the MD simulations, K-edge XAS spectra have been collected for 0.1 M La(NO_3_)_3_ solutions in water and in methanol. First qualitative insight can be gained by comparing the X-ray absorption near edge structure (XANES) part of the absorption spectra, shown in Figure 6A. As can be observed, the two spectra show slight but non-negligible differences in both the amplitude and frequency of the main oscillation after the absorption edge. As it is known, the low energy part of the X-ray absorption spectra is particularly sensitive to the three-dimensional arrangement of the scattering atoms around the photoabsorber due to the higher amplitude of the MS contributions in this energy region.^31,72^ The comparison between the XANES spectra in the two solvents therefore suggests that a different local environment around the La^3+^ ion occurs between the aqueous and methanol solutions. This finding is even more evident from the corresponding Fourier-transforms (FTs) of the absorption spectra calculated in the 3.3–12.3 Å^–1^*k*-range (Figure 6B). The FT first peak position is similar in the two systems, although the intensity is higher in the case of the methanol solution as compared with the aqueous solution one. This observation is compatible with the results of the MD simulations, in particular with the higher total La^3+^ coordination number observed in methanol with respect to the aqueous solution, with the dominating species being a 10-fold complex in the former case (Figure 3A) and a mixture of 9- and 10-fold clusters in the latter (Figure 3B). Note that, in both water and methanol, the La^3+^ ion has been found to be coordinated by oxygen atoms at approximately the same distance (Figure 1) from the MD simulations, in agreement with the FT first peak positions. Conversely, the FTs of the two systems show big differences in the higher distance range. In particular, the methanol solution FT spectrum shows the presence of a peak at about 3.6 Å associated with a second shell contribution, which is not observed in the water FT data (Figure 6B). It is noteworthy that, in previous investigations on the solvation properties of La(NO_3_)_3_ and Ce(NO_3_)_3_ in ethylammonium nitrate, this peak was found to be due to MS effects associated with the coordinating nitrate ligands.^48,66^ The whole picture therefore provides a first experimental confirmation that the nitrate anion is able to enter the La^3+^ ion coordination sphere in methanol solution, at variance with the aqueous one, in agreement with the MD results. Note that other contributions to the FT second-shell peak may arise from the La–C_*MeOH*_ and La–N_*NO*_3_^–^_ paths, which are in any case absent in water.

**Figure 7:**
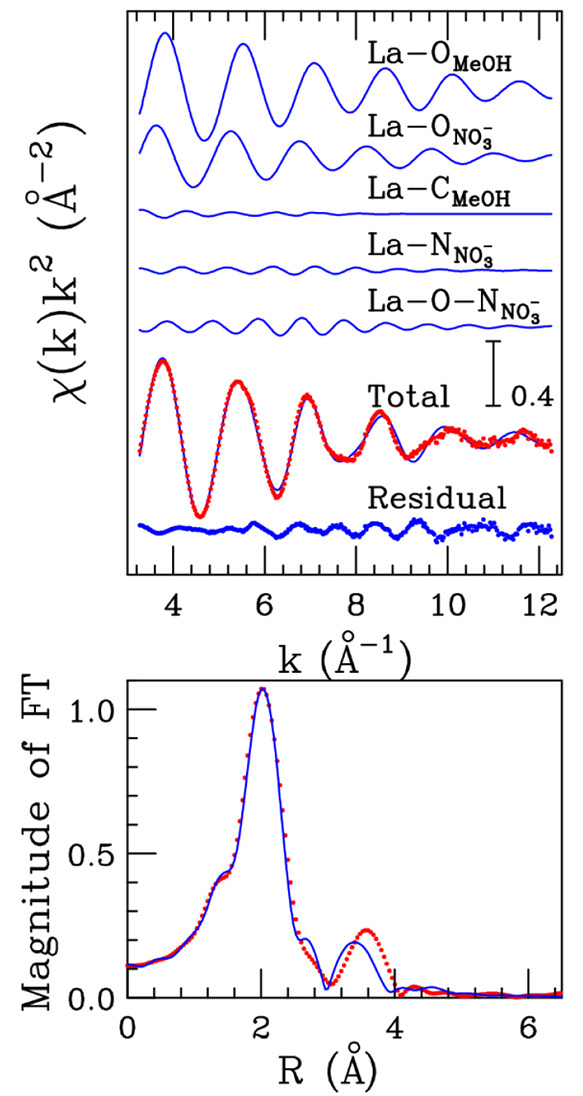
Upper panel: best-fit results for the La K-edge EXAFS spectrum of the 0.1 M La(NO_3_)_3_ methanol solution. From the top the La–O_*MeOH*_, La–O_*NO*_3_^–^_, La–C_*MeOH*_, La–N_*NO*_3_^–^_, and La–O–N_*NO*_3_^–^_ theoretical signals are shown, together with the total theoretical contribution (blue line) compared to the experimental spectrum (red dots), and the resulting residual (blue dots). Lower panel: nonphase shift corrected FT’s of the EXAFS theoretical signals (blue line) and of the experimental data (red dots).

To obtain both definitive proof of the reliability of the MD results and a more quantitative experimental determination of the La^3+^ ion coordination, we analyzed the EXAFS spectrum of the La(NO_3_)_3_ methanol solution. Theoretical two-body signals accounting for the La–O_*MeOH*_ and La–C_*MeOH*_ contributions associated with the coordinating methanol molecules, as well as the La–O_*NO*_3_^–^_ and La–N_*NO*_3_^–^_ signals related to the nitrate anions, have been calculated starting from the structural results of the MD simulations. In addition, to confirm the monodentate coordination of the nitrate anion, an MS signal related to the three body La–O–N_*NO*_3_^–^_ distribution has been calculated with a bond angle of 180°. Least-squares fitting procedures have been performed in the *k*-range 3.3–12.3 Å^–1^, and the best-fit results are shown in the upper panel of Figure 7. As can be observed, the agreement between the theoretical and experimental spectra is very good, and this is also true for the corresponding FT spectra calculated in the *k*-range 3.3–12.3 Å^–1^ (lower panel of Figure 7). The complete list of the optimized structural parameters is reported in Table 2, while the *E*_0_ values resulted in being 3.2 eV above the first inflection point of the experimental spectrum. As can be observed from the structural parameters in Table 2, the number of methanol molecules and nitrate anions coordinating the La^3+^ ion is in excellent agreement with the MD simulation results. In addition, the La–O–N_*NO*_3_^–^_ three-body theoretical signal shows a remarkable amplitude (Figure 7), and this proves both the existence of a monodentate binding mode of the nitrate anion and the sensitivity of the EXAFS spectroscopy toward the fine details of the short distance coordination around the La^3+^ cation. This MS contribution is responsible for the peak at about 3.6 Å in the FT spectrum, and attempts to fit the data without the inclusion of the La–O–N_*NO*_3_^–^_ three-body signal resulted in a remarkably worse agreement between the experimental and theoretical spectra, and the presence of a high frequency contribution in the residual curve. Altogether these results provide a robust experimental confirmation about the ability of the nitrate counterion to enter the La^3+^ solvation sphere in methanol solution and to coordinate the metal in a monodentate configuration, at variance with what happens in aqueous solution.

**Table 2:** Structural Parameters for the La–O_*MeOH*_, La–O_*NO*_3_^–^_, La–C_*MeOH*_, and La–N_*NO*_3_^–^_ Distributions Obtained from the EXAFS Analysis of the 0.1 M La(NO_3_)_3_ Methanol Solution^a^

	*N*	*R* (Å)	σ^2^ (Å^–2^)	β
La–O_*MeOH*_	6.0(3)	2.59(2)	0.006(2)	0.2(2)
La–O_*NO*_3_^–^_	4.0(4)	2.64(2)	0.006(3)	0.4(2)
La–C_*MeOH*_	5.9(4)	3.74(3)	0.011(4)	0.2(2)
La–N_*NO*_3_^–^_	4.9(4)	3.76(3)	0.010(4)	0.1(2)

a *N* is the coordination number, *R* is the average distance, σ^2^ is the Debye–Waller factor, and β is the asymmetry parameter.

## Conclusions

In this work we have carried out a comparative study about the solvation properties of dilute La(NO_3_)_3_ solutions in water and in methanol, with the aim of unraveling the still elusive coordination of the La^3+^ ion in the latter medium. The MD simulations showed that, in methanol solution, the nitrate anions enter the La^3+^ first coordination shell to form inner-sphere complexes. This is at variance with the water case, where the La(NO_3_)_3_ salt is fully dissociated and only water molecules coordinate the metal center. It is noteworthy that the counterions are able to coordinate the metal also in dilute conditions, where the presence of the solvent molecules is overwhelming, suggesting that the affinity of the nitrate anions for the La^3+^ ion is greater in methanol than in aqueous solution. The speciation of the formed solvation complexes shows that the 10-fold coordination is dominating in methanol solution, where an average number of about four nitrate anions is found to coordinate the metal in a monodentate fashion, while the rest of the solvation sphere is completed by methanol molecules to form a global bicapped square antiprism geometry. This picture is different from the aqueous solution, where a more balanced situation is observed between the 9-fold coordination with TTP geometry and the 10-fold one. An experimental confirmation to the MD results has been obtained from the La K-edge XAS data collected on 0.1 M solutions of the La(NO_3_)_3_ salt in the two solvents. The qualitative comparison between the obtained spectra shows clear differences in the XANES region, suggesting the presence of a different local environment around the La^3+^ ion between the aqueous and methanol solutions. This circumstance becomes even more evident from the comparison of the FT spectra, since the methanol solution shows the distinct presence of a second-shell peak between 3 and 4 Å, while this feature is absent in the water case. This peak is associated with the MS effects provided by coordinating nitrate anions and confirms the ability of this counterions to enter the La^3+^ ion first solvation sphere and to coordinate the cation in a monodentate fashion. The fitting of the EXAFS part of the absorption spectrum collected on the methanol solution was carried out starting from the MD results to retrieve a quantitative determination of the La^3+^ ion coordination in this medium. The obtained data unambiguously show that the La^3+^ ion is coordinated both by the nitrate anions and by methanol molecules, confirming the ability of the counterions to form inner-sphere complexes and the structural fashion observed by the MD simulations.

## Supporting Information Available

Pictorial description of the ideal 10-fold bicapped square antiprism polyhedron model (PDF)
