# Supplementary material for: Direct Observation of Contact Ion-Pair Formation in La3+ Methanol Solution
Source: Inorg Chem. 2022 Oct 18;61(43):17313–21. doi: 10.1021/acs.inorgchem.2c02932 (PMC9627567; doi:10.1021/acs.inorgchem.2c02932)
Supplement: Supplementary file 1 — ic2c02932_si_001.pdf [file ic2c02932_si_001.pdf]

Supporting Information

Direct Observation of Contact Ion-Pair Formation in La^{3+} Methanol Solution

Paola D'Angelo*, Valentina Migliorati*, Alice Gibiino, Matteo Busato

[Department of Chemistry, University of Rome "La Sapienza", P.le A. Moro 5, 00185, Rome, Italy

*Corresponding authors

E-mail:

p.dangelo@uniroma1.it

valentina.migliorati@uniroma1.it

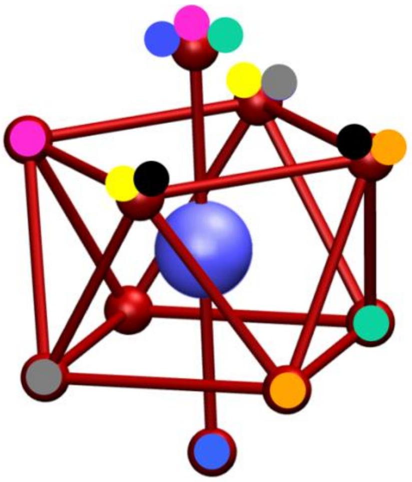

Figure S1. Model of the ideal 10-fold bicapped square antiprism polyhedron showing the pairs of atoms involved, together with the central La^{3+} ion, in the angles calculated in the CDF analysis.
